# Research on improving urban park green space landscape quality based on public psychological perception: a comprehensive AHP-TOPSIS-POE evaluation of typical parks in Jinan City

**DOI:** 10.3389/fpsyg.2025.1418477

**Published:** 2025-02-06

**Authors:** Qingtao Cheng

**Affiliations:** School of Art, Southeast University, Nanjing, China

**Keywords:** urban park, green space, psychological perception, evaluation system, landscape quality

## Abstract

During rapid urbanization, environmental issues significantly affect urban residents health. Urban parks and green spaces play a crucial role in urban planning and layout, significantly impacting residents quality of life and livability. This study constructs a comprehensive landscape evaluation model, “AHP-TOPSIS-POE” from the perspective of behavioral psychological perception. It uses four urban parks in Jinan City (Qianfoshan Park, Baotu Spring Park, Daming Lake Park, and Quancheng Park) as case study samples. This method validates its feasibility by converting subjective perceptions into objective data. The research findings are as follows: (1) Urban park green space landscapes are significantly correlated with public psychological recovery; (2) The weight ranking of the criteria layer is as follows: Landscape Perception (B4) 0.5135 > Social Interaction (B3) 0.3015 > Spatial Form (B2) 0.1244 > Visual Quality (B1) 0.0606; (3) The relative closeness ranking of the four typical urban parks in Jinan City is as follows: Qianfoshan Park > Quancheng Park > Daming Lake Park > Baotu Spring Park. This study aims to reduce the subjectivity of evaluation indicators, raise public awareness of high-quality cognition and emotional experiences, and provide a scientific basis for the development of scientifically reasonable urban park green landscapes.

## 1 Introduction

The rapid urban construction and increasing population density have heightened the demand for urban park green spaces. Urban residents’ focus on urban park green spaces has transitioned from quantity-oriented to prioritizing convenience and accessibility (Zhang et al., 2019). Urban park green spaces, as integral components of urban green infrastructure, not only contribute to creating a conducive living environment but also enhance the psychological well-being of urban residents (Bian et al., 2023). Public psychological perception is the perception resulting from the interaction between individuals and the spatial environment, and it subsequently influences the interaction between individuals and the environment (Huang et al., 2017). Public psychological perception of urban park green spaces encompasses various aspects, including recreation, leisure, aesthetics, sports, education, and spiritual experiences (Huai and Voorde, 2022). Enhancing the quality of urban park green spaces is becoming a strategy to alleviate the diverse mental health challenges faced by urban residents. Hence, investigating the correlation between the provision of urban park green spaces and residents’ mental health is crucial for addressing the imbalance in green space resource allocation and optimizing urban park green space construction (Huang et al., 2017; Huai and Voorde, 2022; Dade et al., 2020). Within the framework of sustainable development, optimizing urban park green spaces is vital for enhancing the psychological well-being of urban residents. Public psychological perception of urban park green spaces is intangible, highly subjective, and non-consumable (Dade et al., 2020). Due to the absence of objective measurement indicators, obtaining precise quantitative evaluation results is challenging (Gai et al., 2022).

**Figure 1:**
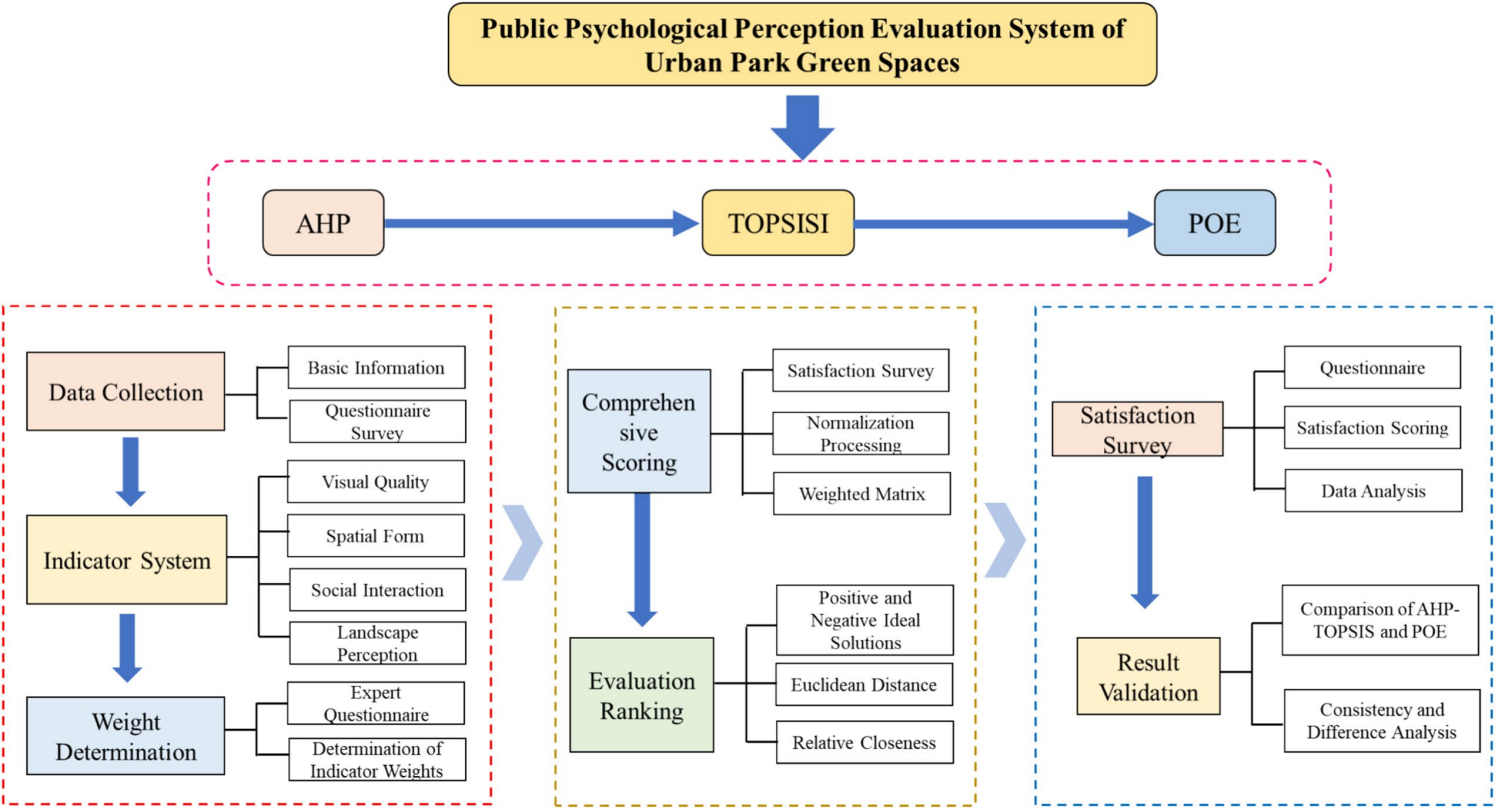
Flowchart of the current study.

In recent years, there has been a growing body of research on urban park green spaces. Internationally, research primarily focuses on urban greening, sustainability, spatial accessibility, environmental justice, and related topics (Kabisch et al., 2015; Li et al., 2016; Sherrouse et al., 2011; Barton and Pretty, 2010). In China, research primarily emphasizes planning layout, ecological benefits, spatial distribution, accessibility, among other aspects (Gupta et al., 2016; YuTing et al., 2021; Clement and Cheng, 2011; Lockwood, 1999). However, research on the impact of urban park green spaces on psychological perception is relatively limited, with most studies focusing on aspects such as their influence on residents’ health, satisfaction, environmental responsibility behavior, among others (Fan et al., 2017; Zhai, 2016; Liu et al., 2021). Quantitative studies on how urban park green spaces affect visitors’ mental health primarily use methods such as the Analytic Hierarchy Process (AHP), fuzzy comprehensive evaluation, scenic beauty evaluation, two-step floating catchment area method, and landscape ecology method (Ma, 2020; Lee et al., 2021; Hagerhall et al., 2004; Dickinson and Hobbs, 2017; Kurt and Douglas, 2013). However, most existing studies rely on either single methods or simple combined models, such as GST + AHP, AHP + TOPSIS, or TOPSIS+POE. While these methods provide insights into the relationship between urban park green spaces and visitors’ mental health, they have certain limitations in terms of comprehensiveness and precision. This study introduces an integrated AHP + TOPSIS+POE model, which combines the advantages of AHP in weight assignment, the strengths of TOPSIS in ranking, and the dynamic feedback mechanism of POE (Post-Occupancy Evaluation). This comprehensive model not only compensates for the limitations of single-method approaches but also provides a more systematic and multidimensional evaluation of how urban park green spaces influence visitors’ mental health. By integrating multiple methodologies, this model offers a more robust and scientific approach to understanding the multifaceted impact of green spaces on psychological well-being. The research framework is depicted in Figure 1.

## 2 Data

### 2.1 Research object

**Figure 2:**
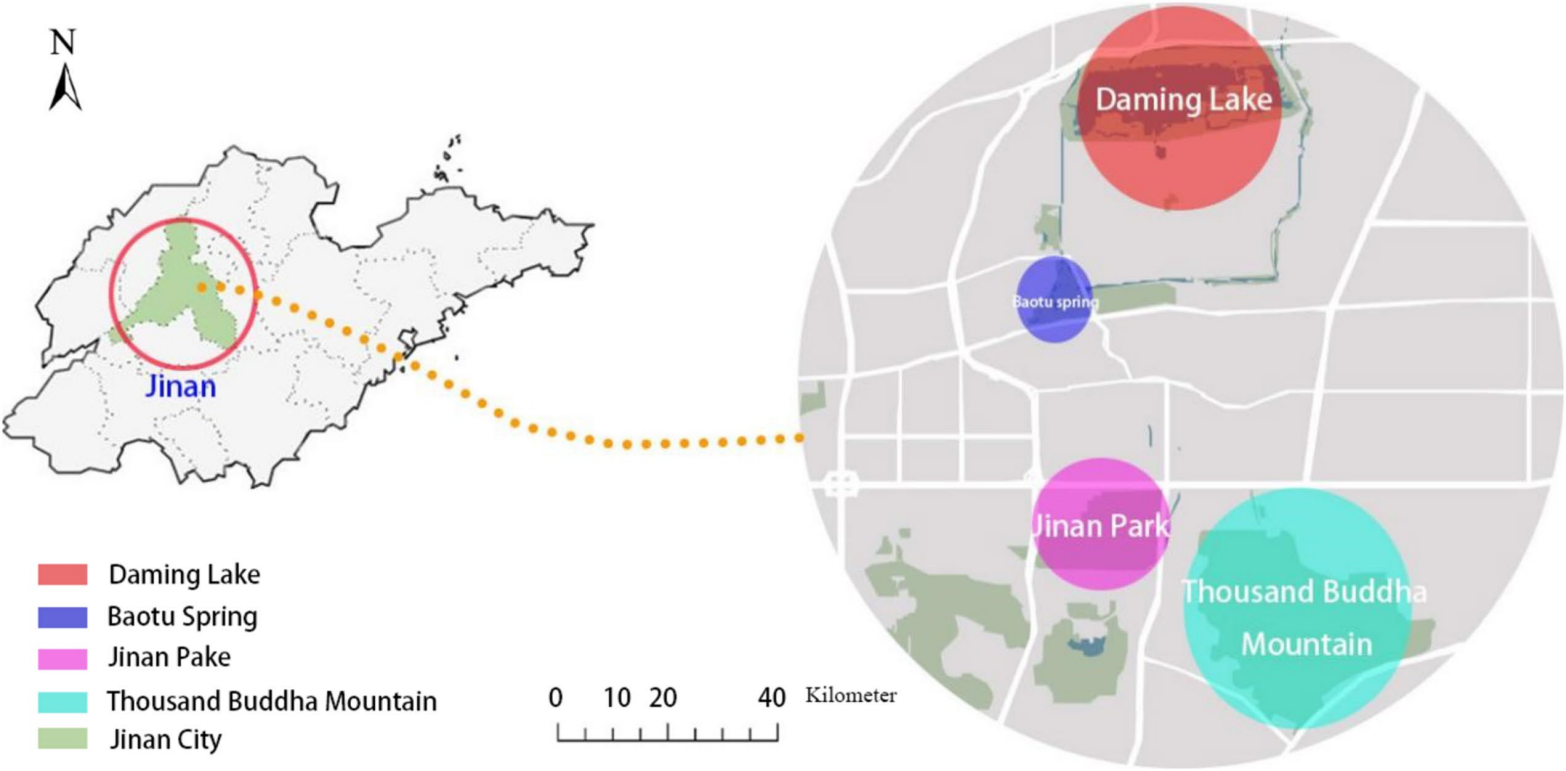
The research area.

The study focuses on four typical urban parks in the main urban area of Jinan: Qianfoshan Park, Baotu Spring Park, Daming Lake Park, and Quancheng Park, with a total area of 164.5 hectares. These parks are mainly categorized as specialized and comprehensive parks. The selection of this study area is based on the following considerations: The selected parks are the major urban parks in Jinan, featuring sufficient visitor flows and abundant blue and green spaces, providing robust samples and research data for analysis. The parks in the study area are primarily positioned based on the cultural services of ecosystems, which are closely related to the psychological perception of urban residents. The selected parks represent the diversity of Jinan’s central urban area in terms of scale, type, and geographic location. Qianfoshan Park is characterized by its mountainous landscape and rich historical and cultural heritage. With high vegetation coverage, it combines natural ecology and cultural landscapes, making it an ideal place for hiking, fitness, and scenic leisure. Baotu Spring Park is renowned for its iconic spring water landscapes and serves as a cultural landmark of Jinan. With its primary focus on sightseeing, it is a representative example of specialized parks. Daming Lake Park is centered around a natural lake, integrating lake wetlands with urban recreational functions. Its distinctive “lake within the city” feature reflects both ecological and cultural value. Quancheng Park, as a modern and comprehensive park, features spacious green spaces and multifunctional areas, catering to the diverse activity needs of citizens. By studying these representative parks, the research aims to capture the impact of urban park green spaces on residents’ psychological perceptions more comprehensively and provide conclusions with broader applicability. The study area is shown in Figure 2.

### 2.2 Index selection and data sources

The selection of indicators is based on the theories of ecosystem services, environmental psychology, and landscape ecology, drawing on the research findings of various experts and scholars regarding the evaluation of urban park green space landscape quality. Additionally, it takes into account the unique landscape characteristics of Jinan’s Qianfoshan Park, Baotu Spring Park, Daming Lake Park, and Quancheng Park. Considering the scientific rigor and applicability of the evaluation system, the framework was constructed from four criteria layers: visual quality, spatial morphology, social interaction, and landscape perception. These four dimensions correspond to the core needs of residents in terms of aesthetics, social interaction, physical perception, and convenience, fully reflecting the psychological impact of urban park landscapes and providing a clear logical foundation for the construction of the evaluation system (Gugulica and Burghardt, 2023; Kaplan and Kaplan, 1989; Guo et al., 2020).

To ensure the scientific, objective, and practical nature of the evaluation indicator system, we invited 18 experts specializing in landscape design from local universities, landscape design institutes, and experienced landscape design enterprises to participate in the construction, selection, and refinement of the indicator system. During this process, the experts, leveraging their extensive professional experience, conducted multiple rounds of discussions and adjustments to an initial pool of 32 evaluation indicators. In the selection process, the experts focused on the scientific validity, applicability, and operability of the indicators. Through in-depth discussions via online and offline meetings, 24 core evaluation indicators were ultimately identified. These indicators comprehensively cover the four criteria layers—visual quality, spatial morphology, social interaction, and landscape perception—forming a scientifically rigorous evaluation system for assessing the psychological perception of urban park green spaces (see Table 1). This system not only reflects the systematic and rigorous nature of theoretical research but also incorporates the practical characteristics of urban parks in Jinan, providing a solid theoretical foundation and strong guidance for subsequent research and practical applications.

**Table 1:** Evaluation indicator system for urban park green space landscape psychological perception.

Objective Layer *A*	Criteria Layer *B*	Indicator Layer *C*	Evaluation Factor Description
Urban Park Green Space Landscape Psychological Perception Evaluation of Four City Parks in Jinan	*B*_1_:Visual Quality	*C*_11_:Vegetation Coverage Rate	Plant density and coverage range
		*C*_12_:Overall Layout of Green Space	Overall planning of green space
		*C*_13_:Terrain and Water Body Design	Layout and form of terrain and water body elements
		*C*_14_:Landscape Diversity	Diverse landscape elements
		*C*_15_:Seasonal Variation of Plants	Changes in plants with seasons
		*C*_16_:Plant Configuration Form	Layout and form of plants
	*B*_2_:Spatial Form	*C*_21_:Spatial Comfort	Comfort brought by landscape space
		*C*_22_:Spatial Privacy	Privacy level of landscape space
		*C*_23_:Spatial Recognizability	Ease of recognizing features and elements in landscape space
		*C*_24_:Sense of Belonging	Sense of belonging for individuals or groups
		*C*_25_:Sense of Identification	Sense of identification elicited in individuals or groups
		*C*_26_:Sense of Familiarity	Degree of familiarity and warmth elicited
		*C*_27_:Sensory Experience	Comprehensive sensory experience through the five senses
	*B*_3_:Social Interaction	*C*_31_:Social Space Design	Design of interactive spaces to promote social interaction
		*C*_32_:Interactive Facilities	Installations and facilities that promote interaction among people
		*C*_33_:Interactive Behavior	Interpersonal interaction and communication behavior
		*C*_34_:Emotional Transmission	Transmission of specific emotions through design and elements
		*C*_35_:Social Atmosphere	Setting of interactive contexts and social nodes
		*C*_36_:Social Activities	Socially oriented activities occurring in the environment
	*B*_4_:Landscape Perception	*C*_41_:Perception of Naturalness	Experience of perceiving the natural environment
		*C*_42_:Perception of Shelter	Perception of shelter and security
		*C*_43_:Perception of Cultural Elements	Experience of perceiving cultural elements
		*C*_44_:Perception of Spatiality	Experience of perceiving the overall spatial layout
		*C*_45_:Perception of Visibility	Perception of open vistas and landscape coherence

## 3 Research methods

### 3.1 AHP theory

The Analytic Hierarchy Process (AHP), proposed by Professor T.L. Saaty of the University of Pittsburgh in the early 1970s, is a decision-making method that converts qualitative problems into quantitative analysis (Wang et al., 2019). It categorizes research objects based on different criteria, dividing them into the goal level, criterion level, and index level. The goal level, which is the highest level, represents the overall objective of the analytic hierarchy. The criterion level and the index level are intermediate steps toward achieving the overall objective, divided into primary and secondary evaluation levels (Weng et al., 2023). Its essence lies in converting the decision maker’s thoughts into a quantifiable model, offering decision-making guidance for complex issues with multiple objectives and criteria.

### 3.2 TOPSIS theory

The Technique for Order Preference by Similarity to an Ideal Solution (TOPSIS) method, also known as the approximation to the ideal solution, ranks evaluation objects by comparing their similarity to an idealized target (Cai et al., 2012). The TOPSIS method calculates the Euclidean distance between each evaluation object and the positive and negative ideal solutions, then computes the relative closeness of each evaluation object to the positive ideal solution. Evaluation objects are ranked in descending order based on their relative closeness, thereby providing a quality ranking. This method is primarily employed in landscape evaluation for urban park quality assessment (Grilli et al., 2020), vegetation greening scheme optimization (Oteros-Rozas et al., 2018), tourism landscape evaluation (Buckland and Pojani, 2023), medical landscape evaluation (Huiyun et al., 2023), etc. A higher relative closeness of a certain urban park to the positive ideal solution indicates that the park exhibits superior landscape quality, nearing the ideal solution.

### 3.3 POE theory

Post Occupancy Evaluation (POE) originated in Western countries in the 1960s (Oteros-Rozas et al., 2018; Buckland and Pojani, 2023). It is a method that tests the rationality of spaces from the perspective of users for spaces currently in use (Wang et al., 2000). To validate the accuracy and practicality of the AHP-TOPSIS evaluation system and mitigate the influence of subjective factors and calculation errors on the results (Zhu et al., 2023), the POE method was employed to conduct a questionnaire survey on the landscape quality of typical urban parks in Jinan City. By statistically calculating the corresponding satisfaction, accurate evaluation criteria for park landscape quality were obtained.

### 3.4 Steps for constructing the AHP-TOPSIS combined model

This study initially employs the Analytic Hierarchy Process (AHP) to compute the weight vectors for each indicator. Subsequently, TOPSIS is utilized to rank various evaluation objects, with the following steps:


$$\omega =\left(\omega_{1},\omega_{2},\cdots ,\omega_{p}\right)\text{,}$$

Satisfy $\overset{n}{\underset{j=1}{\sum }}\omega_{j}=1,\;\omega_{j}\geq 0,\;j=1,2,\cdots ,n$ .

To further utilize TOPSIS for ranking different evaluation objects, the specific steps are as follows:

1. Construct a weighted normalized matrix: Select *n* evaluation objects meeting the criteria, with *P* evaluation indicators. Obtain the original data matrix for scoring, normalize it, and apply weights to obtain matrix *Z*:

$$Z={\left[\begin{array}{cccc} z_{11} & z_{12} & \cdots & z_{1p}\\ z_{21} & z_{22} & \cdots & z_{2p}\\ \cdots & \cdots & \cdots & \cdots \\ z_{n1} & z_{n2} & \cdots & z_{np} \end{array}\right]}_{n\times p}$$

In the equations: $z_{ij}=g_{ij}\times \omega_{j}$ , $g_{ij}$ represents the evaluation value of the *i*-th evaluation object under the *j*-th indicator, it is stipulated that $g_{ij}>0$ , Where $\omega_{j}$ is the weight of the *j-*th indicator, i=1,2,⋯,n , j=1,2,⋯,p .

2. Determine the ideal and negative ideal solutions. Define the ideal solution $M^{+}$ and the negative ideal solution $M^{-}$ based on the maximum and minimum values of each indicator:

$$M^{+}=\left\{{z_{1}}^{+},{z_{2}}^{+},\cdots ,{z_{n}}^{+}\right\},\;{z_{j}}^{+}=\max_{i}z_{ij},\;j=1,2,\cdots ,p\text{,}$$

$$M^{-}=\left\{{z_{1}}^{-},{z_{2}}^{-},\cdots ,{z_{n}}^{-}\right\},\;{z_{j}}^{-}=\min_{i}z_{ij},\;j=1,2,\cdots ,p\text{.}$$

Then, $M^{+}$ represents the ideal vector, and $M^{-}$ represents the negative ideal vector.

3. Calculate the Euclidean distances between each evaluation object and the ideal and negative ideal vectors, denoted as ${d_{i}}^{+}$ and ${d_{i}}^{-}$ respectively:

$${d_{i}}^{+}=\|z_{i}-M^{+}\|=\sqrt{\overset{n}{\underset{j=1}{\sum }}{\left(z_{ij}-{z_{j}}^{+}\right)}^{2}},\;i=1,2,\cdots ,n\text{,}$$

$${d_{i}}^{-}=\|z_{i}-M^{-}\|=\sqrt{\overset{n}{\underset{j=1}{\sum }}{\left(z_{ij}-{z_{j}}^{-}\right)}^{2}},\;i=1,2,\cdots ,n\text{,}$$

where $z_{i}=\left(z_{i1},z_{i2},\cdots ,z_{ip}\right)$ is the *i-*th row of the weighted normalized matrix $Z={\left(z_{ij}\right)}_{n\times p}$ .

4. Calculate the relative closeness ${C_{i}}^{+}$ of each evaluation object to the optimal vector:

$${C_{i}}^{+}=\frac{{d_{i}}^{-}}{{d_{i}}^{+}+{d_{i}}^{-}},\;i=1,2,\cdots ,n\text{.}$$

If $z_{i}=M^{+}$ , then ${C_{i}}^{+}=1$ ; if $z_{i}=M^{-}$ , then ${C_{i}}^{+}=0$ ; ${C_{i}}^{+}$ satisfies $0\leq {C_{i}}^{+}\leq 1$ *f*. Therefore, as ${C_{i}}^{+}$ approaches 1, it indicates that the evaluation object $M_{i}$ is closer to the optimal vector $M^{+}$ .

5. The evaluation objects are sorted in descending order based on the calculated values of relative closeness ${C_{i}}^{+}$ , thus obtaining the ranking results of each evaluation object’s superiority or inferiority.

## 4 Results

### 4.1 Determination of indicator weights using AHP method

Based on the evaluation indicator system, a questionnaire survey was conducted, wherein 18 experts and scholars from Dezhou University, University of Jinan, Shandong Jianzhu University, and other institutions compared the 24 indicators pairwise based on their importance. The judgments of these 18 experts were weighted and averaged to construct five judgment matrices for *B*_1_ ~ *B*_4_, *C*_11_ ~ *C*_16_, *C*_21_ ~ *C*_27_, *C*_31_ ~ *C*_36_, *C*_41_ ~ *C*_45_. Using the eigenvalue method, the weights of criteria layer *B*_1_ ~ *B*_4_ were calculated to be 0.0606, 0.1244, 0.3015, and 0.5135 respectively, along with the weights of indicator layers *C*_1_ ~ *C*_45_ under each criteria layer (see Table 2 for details).

**Table 2:** Weights of criteria layers and indicator layers for each factor.

Objective Layer *A*	Criteria Layer *B*	Weight	Indicator Layer *C*	Weight	Weighted overall ranking
Evaluation of Psychological Perception of Urban Park Green Space Landscape *A*	*B*_1_:Visual Quality	0.0606	*C*_11_:Vegetation Coverage Rate	0.2523	0.0153
			*C*_12_:Overall Layout of Green Space	0.3535	0.0214
			*C*_13_:Terrain and Water Body Design	0.1994	0.0121
			*C*_14_:Landscape Diversity	0.0935	0.0057
			*C*_15_:Seasonal Variation of Plants	0.0408	0.0025
			*C*_16_:Plant Configuration Form	0.0605	0.0037
	*B*_2_:Spatial Form	0.1244	*C*_21_:Spatial Comfort	0.3543	0.0441
			*C*_22_:Spatial Privacy	0.2399	0.0298
			*C*_23_:Spatial Recognizability	0.1036	0.0129
			*C*_24_:Sense of Belonging	0.0312	0.0039
			*C*_25_:Sense of Identification	0.0448	0.0056
			*C*_26_:Sense of Familiarity	0.1587	0.0197
			*C*_27_:Sensory Experience	0.0676	0.0084
	*B*_3_:Social Interaction	0.3015	*C*_31_:Social Space Design	0.3854	0.1162
			*C*_32_:Interactive Facilities	0.2531	0.0763
			*C*_33_:Interactive Behavior	0.6260	0.1887
			*C*_34_:Emotional Transmission	0.0627	0.0189
			*C*_35_:Social Atmosphere	0.0380	0.0115
			*C*_36_:Social Activities	0.0983	0.0296
	*B*_4_:Landscape Perception	0.5135	*C*_41_:Perception of Naturalness	0.1746	0.0897
			*C*_42_:Perception of Shelter	0.0976	0.0501
			*C*_43_:Perception of Cultural Elements	0.0594	0.0305
			*C*_44_:Perception of Spatiality	0.2666	0.1369
			*C*_45_:Perception of Visibility	0.4019	0.2064

To ensure the consistency of the judgment matrices, we calculated the Consistency Ratio (*CR*), where *CR = CI/RI*. Here, *CI* represents the Consistency Index of the judgment matrices, and *RI* represents the Random Index. The *CR* values for matrices *B*_1_ ~ *B*_4_, *C*_11_ ~ *C*_16_, *C*_21_ ~ *C*_27_, *C*_31_ ~ *C*_36_, *C*_41_ ~ *C*_45_ are 0.0351, 0.0255, 0.024, 0.0162, and 0.0117, respectively. All of these values are less than 0.1, indicating that the judgment matrices have passed the consistency test, thereby suggesting that the results of the evaluation index weights are reasonable.

Table 1 displays the descending weights of each criterion in the criterion layer: visual quality (0.0606), landscape perception (0.5135), social interaction (0.3015), and spatial form (0.1244). This suggests that Social Interaction and landscape perception predominantly influence urban park landscape quality. Hence, future urban park planning and design should prioritize enhancing park layout and structure, designing landscape perception effects, and integrating key factors like multifunctional spaces (Woollett and Maguire, 2010; Regina Grazuleviciene et al., 2014). Through optimizing landscape layout and enhancing interactive facilities, improvement in overall landscape quality can be targeted (Yang and Gao, 2022; Xie et al., 2020).

### 4.2 TOPSIS weighted ranking

A satisfaction survey was conducted using the Likert 5-point scale method, focusing on the four major categories and 24 indicators influencing the public’s psychological perception of urban park green space. Respondents rated their satisfaction level for each of the 24 question items on a scale ranging from “very dissatisfied” to “very satisfied, “with corresponding scores of 1 to 5.

Initially, we normalized the collected data and formed the initial matrix. Subsequently, utilizing the weights of each evaluation indicator obtained through the AHP method, we constructed the weighted matrix (refer to Table 3 for specifics).

**Table 3:** Weighted values for the evaluation of public psychological perception in four urban parks in Jinan.

Indicator	Qianfo Shan Park	Baotu Spring Park	Daming Lake Park	Quancheng Park
*C*_11_:Vegetation Coverage Rate	0.00598	0.00309	0.00388	0.00833
*C*_12_:Overall Layout of Green Space	0.01356	0.00918	0.00581	0.01403
*C*_13_:Terrain and Water Body Design	0.00717	0.00346	0.01210	0.00531
*C*_14_:Landscape Diversity	0.00218	0.00088	0.00184	0.00570
*C*_15_:Seasonal Variation of Plants	0.00024	0.00001	0.00112	0.00216
*C*_16_:Plant Configuration Form	0.00157	0.00208	0.00054	0.00163
*C*_21_:Spatial Comfort	0.03574	0.02690	0.00705	0.03319
*C*_22_:Spatial Privacy	0.02103	0.00164	0.00288	0.02646
*C*_23_:Spatial Recognizability	0.00815	0.00570	0.00685	0.00779
*C*_24_:Sense of Belonging	0.00118	0.00149	0.00278	0.00284
*C*_25_:Sense of Identification	0.00273	0.00224	0.00460	0.00376
*C*_26_:Sense of Familiarity	0.00771	0.01704	0.00440	0.00867
*C*_27_:Sensory Experience	0.00240	0.00226	0.00122	0.00821
*C*_31_:Social Space Design	0.09643	0.00526	0.10529	0.08070
*C*_32_:Interactive Facilities	0.04461	0.00980	0.00723	0.07482
*C*_33_:Interactive Behavior	0.04794	0.03370	0.17982	0.13525
*C*_34_:Emotional Transmission	0.01343	0.01318	0.00080	0.00394
*C*_35_:Social Atmosphere	0.00385	0.01123	0.00008	0.00834
*C*_36_:Social Activities	0.00902	0.00012	0.01386	0.02766
*C*_41_:Perception of Naturalness	0.06691	0.03522	0.01061	0.03224
*C*_42_:Perception of Shelter	0.02303	0.05010	0.01602	0.00360
*C*_43_:Perception of Cultural Elements	0.01082	0.02671	0.01473	0.00006
*C*_44_:Perception of Spatiality	0.09771	0.10518	0.01033	0.06535
*C*_45_:Perception of Visibility	0.20640	0.10617	0.03936	0.10684

Subsequently, we determined the positive ideal solution $M^{+}$ and negative ideal solution $M^{-}$ for all indicators regarding the four urban parks, outlined below: $M^{+}$ = {0.00833, 0.01403, 0.01210, 0.00570, 0.00216, 0.00208, 0.03574, 0.02646, 0.00815, 0.00284, 0.00460, 0.01704, 0.00821, 0.10529, 0.07482, 0.17982, 0.01343, 0.01123, 0.02766, 0.06691, 0.05010, 0.02671, 0.10518, 0.20640}, $M^{-}$ = {0.00309, 0.00581, 0.00346, 0.00088, 0.00001, 0.00054, 0.00705, 0.00164, 0.00570, 0.00118, 0.00224, 0.00440, 0.00122, 0.00526, 0.00723, 0.03370, 0.00080, 0.00008, 0.00012, 0.01061, 0.00360, 0.00006, 0.01033, 0.03936}.

Utilizing the provided formulas, we calculate the Euclidean distances ${d_{i}}^{+}$ and ${d_{i}}^{-}$ between each of the four city parks and the positive $M^{+}$ and negative ideal solutions $M^{-}$ , respectively. A smaller value of ${d_{i}}^{+}$ signifies that the park is closer to the positive ideal solution, indicating a higher level of landscape quality. Conversely, a smaller value of ${d_{i}}^{-}$ suggests that the park is closer to the negative ideal solution, implying a lower level of landscape quality. Additionally, we determine the relative closeness degree ${C_{i}}^{+}$ for each park to the positive ideal solution. A value closer to 1 for ${C_{i}}^{+}$ signifies that the park is nearer to the positive ideal solution $M^{+}$ . Based on these calculations, the parks are ranked as follows (see Table 4). Qianfoshan Park (0.61397), Quancheng Park (0.56477), Daming Lake Park (0.44923), and Baotu Spring Park (0.37774). This ranking reflects each city park’s performance relative to the positive ideal solution, with higher values indicating closer proximity to the positive ideal solution.

**Table 4:** Discrepancy in landscape quality among four urban parks in Jinan and their alignment with positive/negative ideal solutions.

	Qianfoshan Park	Baotu Spring Park	Daming Lake Park	Quancheng Park
Euclidean Distance to Positive Ideal Solution ${d_{i}}^{+}$	0.14153	0.21976	0.21935	0.13561
Euclidean Distance to Negative Ideal Solution ${d_{i}}^{-}$	0.22510	0.13340	0.17891	0.017598
Relative Closeness Degree *C_i_*^+^	0.61397	0.37774	0.44923	0.56477
Ranking	1	4	3	2

Following a comprehensive evaluation of the four urban parks in Jinan, Qianfoshan Park exhibits the highest landscape quality assessment, whereas Baotu Spring Park shows relatively poorer performance. Parks with superior landscape quality should concentrate on fortifying and enhancing their current strengths, delving into their distinctive features, and advancing the landscape quality of city parks further (Jiang et al., 2022). Conversely, addressing deficiencies, resolving extant issues, and fostering quality enhancement are paramount for parks with lower landscape quality evaluations, aiming for holistic development in all aspects.

Qianfoshan Park demonstrates superior visual quality attributed to its well-executed overall layout, topography, water body design, and vegetation coverage, indicating a higher landscape quality. Its spatial form provides commendable comfort and privacy, enhancing visitors’ sense of security, mitigating noise disturbances, and fostering psychological well-being. Notably, Qianfoshan Park excels in social interaction with its well-designed social spaces, interactive facilities, and engaging elements, fostering a vibrant and socially rewarding environment that bolsters interpersonal relationships. Its distinctive visual and spatial aspects contribute to a favorable perception of the park (Vîlcea and Șoșea, 2020). Conversely, Baotu Spring Park exhibits deficiencies in landscape quality assessment, necessitating improvements in plant arrangement and seasonal landscape changes to enhance overall visual appeal. Enhancements in spatial form should address privacy concerns and foster a sense of belonging. Promoting social interaction may require the introduction of appealing social spaces and activities to encourage park visitors’ interaction. Emphasizing the integration of historical and cultural elements can enhance the park’s uniqueness and attractiveness.

Overall, evaluating the public’s psychological perception of urban park green space landscapes allows for a more nuanced understanding of each park’s strengths and weaknesses, enabling the provision of guidance and recommendations for enhancing the landscape quality of urban parks in future planning endeavors. These analytical findings facilitate targeted design interventions and improvements aimed at achieving comprehensive enhancements in the landscape quality of urban park green spaces (Deng et al., 2019). The final ranking indicates that Qianfoshan Park leads in landscape quality, followed by Quancheng Park and Daming Lake Park, while Baotu Spring Park requires further enhancement.

### 4.3 POE method survey verification

In order to validate the accuracy and rationality of the evaluation system while mitigating the influence of confounding factors and computational errors on the evaluation outcomes, we conducted a Post-Occupancy Evaluation (POE) questionnaire survey among users of the four city parks. Subsequently, we statistically analyzed the questionnaire responses to ascertain satisfaction levels regarding landscape quality.

The survey questionnaire comprised two sections: one focused on gathering respondents’ basic information, while the other assessed satisfaction with the urban park green space landscape psychological perception (see Table 5). Using the Likert 5-point scale, we delineated specific options in the satisfaction survey, categorized into five levels: extremely satisfied, satisfied, average, dissatisfied, and extremely dissatisfied. Each category was assigned values ranging from 1 to 5 based on the relative importance of different evaluation factors. Respondents were instructed to rate their satisfaction with various indicators. The satisfaction score for each evaluation factor was computed as the arithmetic mean of the scores assigned to its constituent indicators, with the superior indicator’s satisfaction equivalent to the average satisfaction score of its subordinate indicators.

**Table 5:** Urban park green space landscape perception satisfaction evaluation system.

Overall objective	Evaluation aspect	Evaluation element
Urban Park Green Space Landscape Pschological Perception Satisfaction	Visual Quality	Vegetation Coverage Rate
		Overall Layout of Green Space
		Terrain and Water Body Design
		Landscape Diversity
		Seasonal Variation of Plants
		Plant Configuration Form
	Spatial Form	Spatial Comfort
		Spatial Privacy
		Spatial Recognizability
		Sense of Belonging
		Sense of Identification
		Sense of Familiarity
		Sensory Experience
	Social Interaction	Social Space Design
		Interactive Facilities
		Interactive Behavior
		Emotional Transmission
		Social Atmosphere
		Social Activities
	Landscape Perception	Perception of Naturalness
		Perception of Shelter
		Perception of Cultural Elements
		Perception of Spatiality
		Perception of Visibility

**Figure 3:**
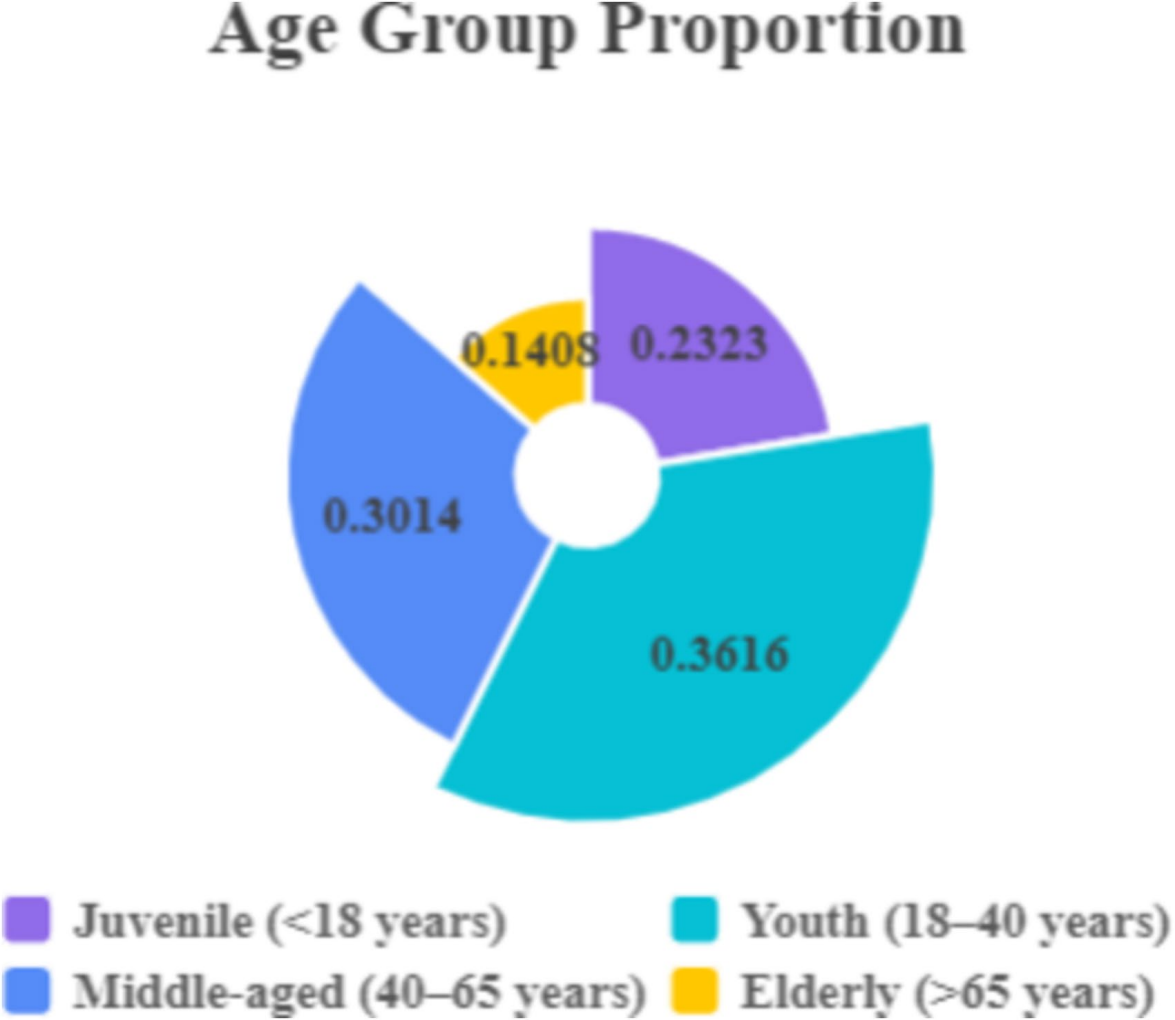
Age group proportion of respondents in the four urban parks.

**Figure 4:**
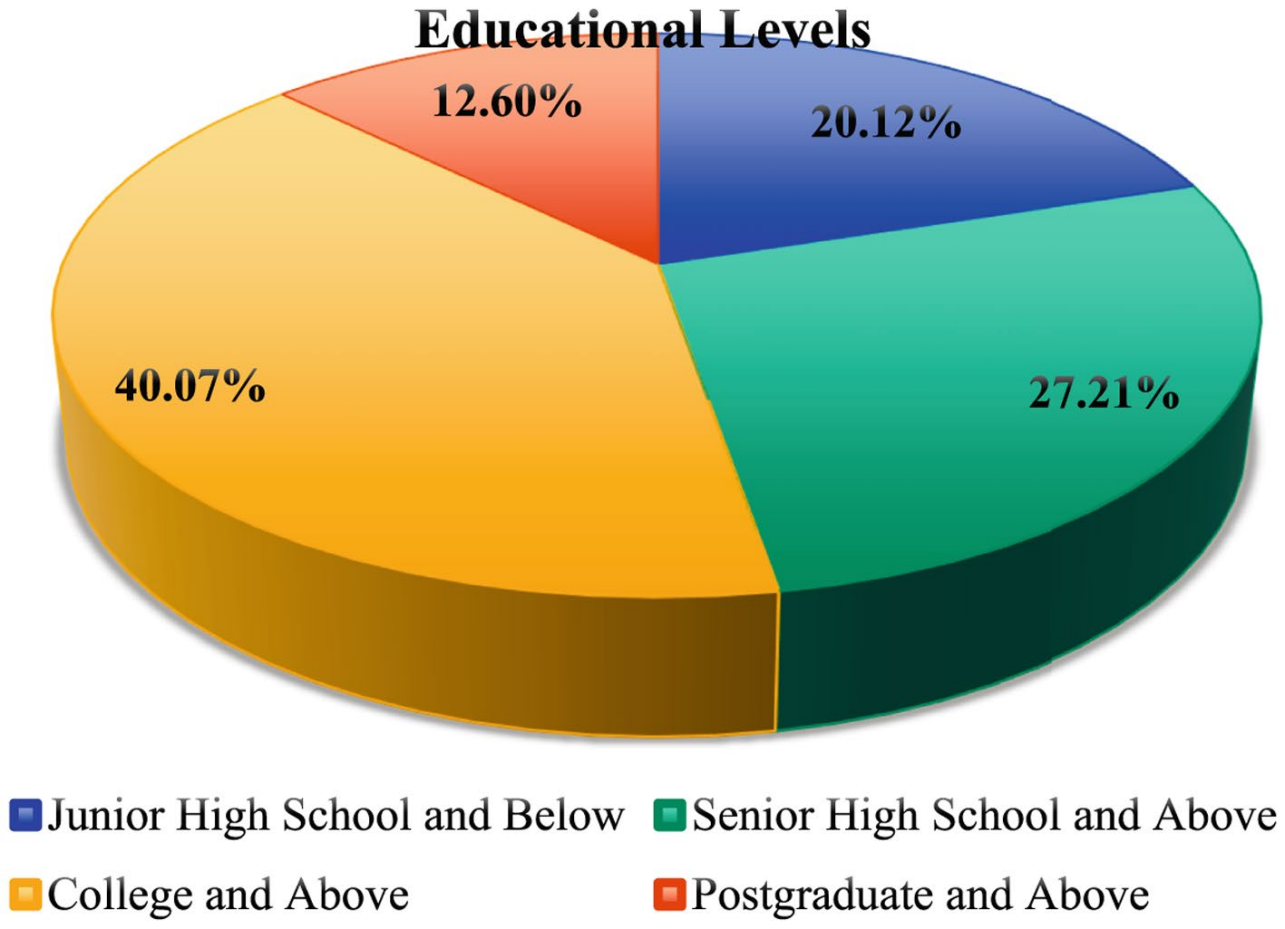
Educational level proportion of respondents in the four urban parks.

To ensure the accuracy of the evaluation process, questionnaire surveys were conducted at the four city parks from August 14 to 21, 2022, and from March 1 to 7, 2023. A total of 1,200 questionnaires were distributed across the parks, yielding 1,088 valid responses, with a response rate of 90.67%. The respondents represented four age groups: children (<18 years old), youth (18–40 years old), middle-aged (40–65 years old), and elderly (>65 years old), comprising 23.23, 36.16, 30.14, and 14.08% of the sample, respectively, as shown in Figure 3. Educational backgrounds varied, with 219 respondents holding a junior high school education or below (20.12%), 296 respondents having a college education or above (27.21%), 436 respondents possessing a junior college education or above (40.07%), and 137 respondents holding a graduate-level education or above (12.60%), as shown in Figure 4.

The satisfaction scores of the surveyed items ranged from 1 to 5, where a score of 5 denoted extreme satisfaction, scores between 3 and 4 indicated satisfaction, a score of 3 indicated an average sentiment, scores between 2 and 3 reflected dissatisfaction, and scores between 1 and 2 signified extreme dissatisfaction. Through the computation of satisfaction scores, we can ascertain the rationality and feasibility of the evaluation system.

The analysis reveals that Qianfoshan Park has the highest satisfaction ranking, followed by Quancheng Park, Daming Lake Park, and Baotu Spring Park, in descending order. The calculated landscape satisfaction rankings of Jinan’s urban parks (refer to Table 6) are consistent with the evaluation rankings generated by the AHP-TOPSIS combined evaluation model, offering robust validation for the evaluation model and its findings.

**Table 6:** Survey results of landscape satisfaction in four urban parks in Jinan.

Evaluation object	Survey total/unit	Extremely satisfied/unit	Satisfied/unit	Moderate/unit	Dissatisfied/unit	Extremely dissatisfied/unit	Satisfaction rate
Qianfoshan Park	285	214	32	22	13	4	86.32%
Quancheng Park	267	176	31	28	25	7	77.53%
Daming Lake Park	273	164	47	38	19	5	77.30%
Baotu Spring Park	263	142	36	53	28	4	67.69%
Total	1,088	696	146	141	85	20	77.39%

## 5 Discussion

The psychological perception evaluation of urban park green space is a comprehensive evaluation problem involving multiple factors, characterized by certain fuzziness and complexity, which makes it difficult to fully quantify. This study effectively addresses this issue by constructing a combined AHP-TOPSIS-POE evaluation model.

Firstly, the AHP method was employed to analyze the psychological perception evaluation index system of urban park green space, determining the weights of the criteria layer and indicator layer, thus providing a quantitative basis for evaluation. Secondly, the TOPSIS method was used to calculate the relative closeness of each evaluation object to the positive ideal solution, ranking the landscape quality of four typical urban parks in Jinan City. Lastly, the POE method was applied to validate park landscape satisfaction through surveys. The results showed that the POE outcomes were generally consistent with the rankings derived from the AHP-TOPSIS model, which to some extent verifies the accuracy and scientific validity of the AHP-TOPSIS-POE evaluation model. This provides a reliable basis for landscape optimization and design of urban parks.

### 5.1 Comparative analysis of AHP-TOPSIS and POE results

Although the evaluation results of AHP-TOPSIS and POE are consistent in terms of overall ranking, there are certain differences in specific details. For example, the POE satisfaction scores for Daming Lake Park and Quancheng Park are 77.53 and 77.30%, respectively, which are almost identical. However, in the AHP-TOPSIS evaluation results, the relative closeness of Daming Lake Park to the positive ideal solution is significantly lower than that of Quancheng Park. These differences may be related to the following factors:

#### 5.1.1 Influence of weighting

The AHP-TOPSIS model relies on the weights assigned by experts, where high weights for “Landscape Perception” (B4 = 0.5135) and “Social Interaction” (B3 = 0.3015) may have contributed to Quancheng Park’s relatively higher scores. In contrast, the POE method directly reflects the subjective satisfaction of the public, which may place more emphasis on intuitive perception indicators such as “Visual Quality” and “Spatial Form,” thereby narrowing the satisfaction gap between Daming Lake Park and Quancheng Park.

#### 5.1.2 Differences between model-based and subjective perceptions

The AHP-TOPSIS calculation is based on structural correlations between indicators and assigned weights, emphasizing the scientific rationality of the theoretical framework and the logical consistency of data. In contrast, the POE method focuses on user experiences and subjective perceptions, which may be influenced by individual preferences. For instance, Daming Lake Park, characterized by its “city-lake” landscape, may generate higher public satisfaction in aspects such as “environmental belonging” or “hydrophilicity,” which might be underrepresented in the comprehensive scoring of the AHP-TOPSIS model.

#### 5.1.3 Variations in data sources

The AHP-TOPSIS model relies on expert opinions and the indicator system, while POE data is derived directly from questionnaire surveys involving diverse public groups of different ages, education levels, and places of residence. This makes POE results more reflective of the public’s true feelings. Thus, the combination of subjective and objective evaluation methods effectively compensates for the limitations of using a single evaluation approach.

### 5.2 Directions for improvement

Although the AHP-TOPSIS-POE evaluation model successfully addresses the quantification challenge in psychological perception evaluation of urban park green spaces, there is still room for improvement:

#### 5.2.1 Further optimization of weight distribution

In this study, the indicator weights were mainly determined by expert scoring. Although consistency testing was passed, there may still be subjective bias in expert opinions. Future research can incorporate more public perception-based weight allocation methods (e.g., principal component analysis or fuzzy comprehensive evaluation) to enhance the objectivity of the evaluation system.

#### 5.2.2 In-depth exploration of public perspectives

Although the POE method provides direct evaluations of landscape quality from the public, its data analysis has yet to delve deeply into the perceptual differences among various groups (e.g., local residents vs. non-local visitors, different age groups, and varying educational levels). Future research could refine data analysis methods for public perception to provide more targeted landscape optimization recommendations for different groups.

#### 5.2.3 Inclusion of more park samples

This study focuses on four typical parks in Jinan City. Although the sample has a certain degree of representativeness, it cannot fully cover the characteristics of different types of urban parks. Future studies could expand the sample scope to include more specialized and comprehensive parks, thereby improving the universality and applicability of research conclusions.

## 6 Landscape quality enhancement strategies

By constructing multi-dimensional and interconnected activity spaces, optimizing landscape sequences and spatial hierarchies, enhancing interactive landscape quality, and strengthening cultural and ecological values, the landscape quality of urban parks in Jinan City will be significantly improved (He et al., 2020). Based on the targeted strategies proposed in this study, urban parks can not only better meet residents’ psychological and perceptual needs but also play a more important role in enhancing urban quality of life and optimizing the urban ecosystem. These strategies provide a scientific foundation for the future planning and design of urban parks, contributing to the high-quality development of Jinan City’s urban green space system.

### 6.1 From single to multi-dimensional: constructing diverse and integrated Urban Park spaces

High-quality urban park landscapes can provide residents with diverse activity spaces. Jinan City’s urban parks need to achieve multi-dimensional space construction based on single-function foundations, creating comprehensive material space layers for residents’ free living in the form of “park +.” Studies show that “landscape perception” and “social interaction” have higher weights in the quality of urban park landscapes, indicating that urban parks need to further enhance residents’ psychological satisfaction by optimizing diversified activity spaces and increasing functional complexity (Zhou et al., 2023). By improving pedestrian paths, bicycle lanes, and public transportation connections, a “fully circulated” park grid system can be constructed, making it easy for residents from different areas to access the parks. Combining leisure, sports, education, and entertainment to form multi-functional spaces that satisfy individual activities and are suitable for social interactions. At the same time, focusing on the combination of ecological functions of green spaces and humanized needs to promote diversification and comprehensiveness of park spaces, providing residents with a “suitable for both activity and relaxation” spatial experience (Huang et al., 2008). Appropriately introducing drought-resistant plant species to enhance ecological functions, while combining pedestrian walkways and shading designs to meet residents’ comfort needs, collaboratively promoting the physical and mental health and spiritual life of urban residents, making urban parks truly serve as the “living rooms” of the city.

### 6.2 From static to dynamic: optimizing landscape sequences and spatial hierarchies of urban parks

Although visual quality has a lower weight in the landscape quality of Jinan’s urban parks, it still plays an important role in enhancing residents’ aesthetic pleasure and sense of belonging, especially in parks with strong cultural and scenic appeal (such as Baotu Spring Park). Optimizing the landscape sequences and spatial hierarchies of urban parks can guide residents to experience park activities more deeply and enhance their psychological perception effects (Alessa et al., 2008). By introducing seasonally changing plants and landscape elements, parks can display different charms in different seasons. For example, adding seasonal plant changes and topographical designs in Qianfoshan Park to enrich the layers of visual perception (Hao et al., 2021). Combining the “urban lake” feature of Daming Lake Park, by planning open vistas and hierarchical landscape belts, creating more coherent and rhythmically distinct landscape sequences, increasing residents’ overall environmental identification. Introducing more design elements that integrate spring water culture in Baotu Spring Park, creating landscape forms that combine cultural and scenic appeal, making its landscape layers more abundant.

### 6.3 From perception to interaction: enhancing the interactive landscape quality of urban parks

Social interaction is an important component of public psychological perception, and optimizing interactive spaces helps improve the overall landscape quality of parks. Appropriate spatial scales and humanized interaction designs can effectively stimulate residents’ sense of participation and belonging, enhancing mental health and happiness (Shi et al., 2023). Reasonably dividing static rest areas, dynamic activity zones, and open social spaces, controlling the degree of spatial enclosure and scale. Designing more inclusive and flexible interactive venues for Quancheng Park to attract more citizens to participate. Adding facilities suitable for different age groups (such as children’s playgrounds and elderly fitness areas) to meet diverse usage needs, while adding fun and interactivity to the parks; arranging plants at varying heights and reasonably distributing landscape nodes to provide residents with psychological safety and a sense of domain, such as appropriately increasing sheltered spaces in Daming Lake Park to optimize the balance between privacy and openness (Wang et al., 2021). Introducing interactive designs for walkways and hiking paths in Qianfoshan Park, leveraging the advantages of the natural environment to encourage slow-paced activities, enhancing residents’ physical and mental health experiences.

### 6.4 From general to distinctive: strengthening the cultural and ecological values of parks

In Jinan’s urban park evaluation index system, landscape perception has the highest weight, with natural perception, cultural perception, and spatial perception being important sub-indicators (Yang et al., 2023). Therefore, urban parks should focus on exploring unique cultural elements and enhancing natural landscapes to improve the overall attractiveness of the parks and residents’ psychological identification. In Baotu Spring Park and Daming Lake Park, strengthen the interpretation of spring water culture and historical culture by introducing iconic sculptures, cultural corridors, and other designs to enhance cultural perception. By optimizing vegetation coverage and the overall layout of green spaces, improve residents’ satisfaction with natural perception. In Quancheng Park, increase open green spaces and natural landscape nodes to enhance the environment’s biodiversity and visual attractiveness. Optimize the design of visual perception and spatial perception within the parks, creating an interconnected green network system, so that residents can gain a higher sense of overall presence and belonging within the parks.

## 7 Conclusion

This study conducted a comprehensive evaluation of the psychological perception of green landscapes in four typical urban parks in Jinan City using the AHP-TOPSIS-POE evaluation model, providing valuable insights. The findings not only validate the scientific and practical applicability of the evaluation model but also offer references for the planning and design of urban park landscapes. However, there are certain limitations in this study that need to be addressed in future research:

### 7.1 Research limitations

The evaluation criteria and weights were determined by experts and scholars, which might introduce subjective biases and fail to fully capture public perceptions and preferences. The study data were based solely on four typical urban parks in Jinan City, resulting in a relatively limited sample size, which may restrict the generalizability of the findings. Additionally, the results are based on data collected at specific time points and do not reflect the evolution of public psychological perceptions over different time periods.

### 7.2 Future research directions

Future research should explore incorporating public opinions and preferences into the evaluation criteria alongside expert weights, using methods such as surveys or interviews to enhance the objectivity and representativeness of the evaluation system. It is recommended to expand the study scope to include more urban parks in different geographical regions, covering various types and scales of parks, to improve the generalizability of the findings (Muhamad Nor et al., 2024). Longitudinal data collection across multiple time points should be conducted to examine the long-term impact of changes in urban park management, environmental conditions, and socioeconomic factors on public psychological perceptions. Furthermore, integrating technologies such as remote sensing, geographic information systems (GIS), and virtual reality could provide more precise and comprehensive data support for the evaluation of urban green spaces. The application of these technologies will further enhance the accuracy, efficiency, and practicality of the evaluation model.

## Data Availability

The original contributions presented in the study are included in the article/supplementary material, further inquiries can be directed to the corresponding author.
